# Study of Influence of Atmospheric Conditions on the Thermal Properties of Sleeping Bags

**DOI:** 10.3390/ma15061992

**Published:** 2022-03-08

**Authors:** Ewa Skrzetuska, Michał Agier, Izabella Krucińska

**Affiliations:** Institute of Material Science of Textiles and Polymer Composites, Faculty of Material Technologies and Textile Design, Lodz University of Technology, 116 Żeromskiego Street, 90-924 Lodz, Poland; m.agier@outlook.com (M.A.); izabella.krucinska@p.lodz.pl (I.K.)

**Keywords:** thermal mannequin, thermal insulation, sleeping bag, CAD modelling, simulations, the finite volume method

## Abstract

The thermal properties of clothing products are influenced by external environmental parameters, such as temperature, humidity, air flow and parameters related to the user’s body, which mainly include temperature and humidity. Depending on the type of raw material, its thickness and the material manufacturing technique, clothing products are characterised by certain insulating properties to protect the human body from external factors. A multilayer system made of different material groups can change the thermal insulating capacity significantly, which cannot be determined by the testing of individual layers used in the production. In order to determine the influence of weather conditions on thermal insulation and air permeability, tests were carried out for two types of sleeping bags (summer and autumn) produced by the same manufacturer, differing in insulation thickness. Simulations were carried out using SolidWorks and verified using a Newton thermal mannequin. During tests, both the temperature (range from −20 °C to 20 °C) and humidity values were changed (range 40–80% humidity). For sleeping bags, the effective thermal insulation decreases along with the increase of temperature and decrease of humidity. It can be observed, for the autumn sleeping bags, that for a temperature of 20 °C and humidity of 60%, the thermal insulation is 1.063 m^2^·K·W^−1^, while for a temperature of −20 °C and humidity of 60% thermal insulation increases significantly and amounts to 1.111 m^2^·K·W^−1^. A similar situation occurs for the effective thermal insulation of a summer sleeping bag (20 °C/60% thermal insulation is 0.794 m^2^·K·W^−1^, while for −20 °C/60%—0.851 m^2^·K·W^−1^. During the tests, the humidity and temperature between the layers of the clothing system were also controlled, in order to learn more about the influence of these parameters on the thermal insulation properties of the sleeping bags.

## 1. Introduction

Sleeping bags are products made of textile materials, which are intended to provide the user with protection against the adverse effects of the external environment and the effects of hypothermia during sleep in changing climatic conditions of the environment. The main function of a sleeping bag is to protect the user’s body and to provide physiological comfort during use. The feeling of physiological comfort is a complicated issue due to the multitude of factors affecting its value, as well as due to the subjective feeling of warmth by the user [1,2,3,4]. An important concept is the heat balance equation, which includes several factors affecting heat exchange between the user’s body and the external environment. This concept is related to the intensity of energy expenditure of the body in order to maintain a constant body temperature. The human body functions in a variable environment, and its heat balance is influenced by various parameters of the external environment—air temperature, humidity, water vapour pressure and air flow velocities as well as thermal insulation and water vapour resistance of clothing. All these parameters affect the amount of heat lost by the body and the homeostatic processes, which are designed to maintain the internal body temperature at 36.5 °C ± 3 °C [4,5,6]. 

In order to maintain this balance, a thermal equilibrium must be maintained between the endogenous heat of our body and the exogenous heat that our body obtains from outside. This state can be represented by the user’s heat balance Equation (1) [7], which takes the form according to Annex C in EN ISO 23537:

(1) $$M=M_{B}+M_{S}$$

(2) $$M_{B}+M_{S}={\;H}_{c}+H_{e}+H_{\mathrm{res}}+\Delta S$$

where M [W·m^−^^2^] is the metabolic heat produced by the body of the sleeping bag user (2). It consists of the total heat energy produced by the human metabolism (M_B_—basic metabolic heat produced in recumbent position) as well as additional metabolic heat caused by phenomena, such as shivering—M_S_ [8]. H_c_ [W**·**m^−^^2^] is the dry heat flux emitted by the body, which enters the external environment through uncovered parts of the user’s body or flows through the surface of the sleeping bag. Assuming that the atmosphere is constant, the dry heat flux can be calculated by dividing the air temperature difference (t_S_ [°C]) by the temperature of the user’s skin (t_SK_ [°C]) by the effective heat resistance of the sleeping bag—R_c,eff_ [m^2^·K·W^−1^]; ∆S—is the change in internal body temperature of the sleeping bag user over the duration of the test, expressed as [W**·**m^−^^2^] [8]:

(3) $$H_{C}=\frac{\left(t_{\mathrm{sk}}-t_{s}\right)}{R_{c,\mathrm{eff}}}$$

He, on the other hand, corresponds to the heat loss due to evaporation of perspiration from the skin surface of the user, expressed as [W∙m^−2^]. According to the mentioned standard calculations, this unit uses the equation [8]:

(4) $$H_{e}=\frac{w\left(p_{\mathrm{sd}}-p_{a}\right)}{R_{e,\mathrm{eff}}}$$

where w—skin perspiration—is the part of the skin that is exposed and is involved in sweat evaporation. A constant value is assumed for resting activity in cold conditions, and this is 6%; p_sd_—partial pressure of water vapour on wet skin, expressed as [Pa]; p_a_—partial pressure of water vapour in the air, expressed as [Pa; R_e,eff_—effective water vapour resistance in the sleeping bag [m^2^·Pa·W^−1^]; H_res_ [W**·**m^−^^2^] is the heat loss through the breathing system of the sleeping bag user [8].

(5) $$H_{\mathrm{res}}=M\cdot \left[0.5524-0.00144\cdot \left(t_{a}+273\right)-0.00632\cdot p_{a}/\left(t_{a}+273\right)\right]$$

where M—metabolic heat produced [W**·**m^−^^2^]; t_a_– ambient air temperature [°C]; p_a_—partial pressure of water vapour in the air, expressed as [Pa] [8].

The correct choice of the material composition of sleeping bags is a very important issue to ensure an adequate level of protection when staying in a low temperature environment. A multilayer material system, such as a sleeping bag or clothing system, is a barrier to the transport of heat and water vapour from the skin to the environment. 

Equations (3) and (4) indicate that the thermal balance behaviour of the users of sleeping bags depends on their effective thermal and water vapour resistance of the layering system of the sleeping bags together with the air layer contained in the space between the sleeping bag and the user’s body. Of great importance, as well, is the volume of air between the layers of the sleeping bag material systems and the boundary layer of air in contact with the outer surface of the material system and the atmosphere of the external environment. One of the primary functions of textile products is thermal insulation. This is their ability to exchange heat between the body and the external environment. The rate and manner of heat exchange depends on the insulating properties of the textiles and the insulation and thermal conductivity of each layer of the clothing in the tested system [9,10,11]. The values of the mentioned parameters can be determined using the test method with a thermal mannequin in accordance with PN-EN ISO 23537-1:2017-02.

Due to the growing interest in spending time outdoors and mountain expeditions, there has been a significant increase in the consumption of sleeping bags. Mountain hiking and climbing are a popular form of spending an active holiday. The use of different material arrangements in sleeping bags has increased in recent years, allowing them to be used in a variety of conditions while maintaining comfort. The use of the most modern textile science and technology in the production of this type of materials is developing every day [12,13,14]. Winter sleeping bags must provide adequate thermal insulation, as well as protection against weather conditions, while summer sleeping bags must—oppositely—provide lower thermal insulation; therefore, different composition of raw materials should be considered.

Many previous studies focused on the study of thermal properties in single and double-layer structures with variable raw material compositions to achieve the optimal level of comfort [12,15,16,17,18,19,20].

The aim of the presented paper was to present a study on the thermal insulation of two types of sleeping bags, summer and autumn, in different temperature conditions in order to determine the influence of the external conditions on the obtained result of thermal insulating power. The novelty of the article lies in conduction of the thermal insulation tests in changing climatic conditions, which, so far, has not been conducted in such a wide range. The research was conducted in conditions of −20 °C, −10 °C, 0 °C, 10 °C and 20 °C. Separate temperature and humidity sensors were used to record the climatic conditions in different parts between the sleeping bag and user. In addition, the tests were preceded by computer simulations in terms of temperature changes in individual layers of the sleeping bag.

## 2. Materials and Methods

### 2.1. Research Material

One set of sleeping bags for military use was used for the research. It consisted of two sleeping bags: a summer sleeping bag with less filling and an autumn sleeping bag with a much higher content of insulating fibres. Both sleeping bags of the mummy type were made of the same polyamide material in khaki colour with additional waterproofing. The internal filling consisted of two layers of non-woven polyester fabrics. They differed in the weight and functional elements—the autumn sleeping bag had a shorter zip due to which there are no possible gaps in feet and calves causing the cold air to flow inside the textile system. The summer sleeping bag, on the other hand, has a longer zipper running the length of the product, allowing the sleeping bag to be wide open and has a mosquito net hidden in a pocket in the hood of the product. Table 1 compares the weights of the sleeping bags and the surface weights of all the textile materials used for their construction.

Apart from the two sleeping bags and the mannequin, reference clothing made of knitted polyester was used in the research, which represented the clothing worn under the sleeping bags by the users. The set of reference clothing consisted of sweatshirt and trousers, made of the same polyester-knitted fleece type, in addition to which cotton socks were put on the mannequin. The entire set-up fulfils the parameters for type B reference clothing provided in EN 342:2018-01.

### 2.2. Research Methodology

#### 2.2.1. Model Designing

**Figure 1:**
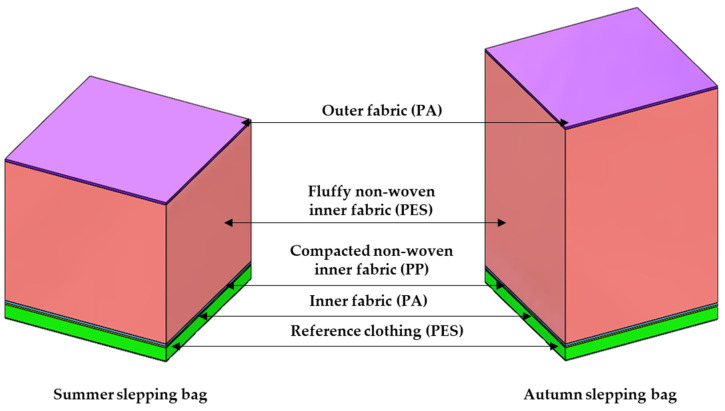
3D models of sleeping bags.

The three-dimensional geometric models of the two-tested material arrangement of sleeping bags were designed using SolidWorks CAD software [21]. First, 3-D models of all five textiles, making up layers of the sleeping bags, were designed and reference clothing: (1) outer fabric-PA fabric, khaki dyed with panther print and waterproof finish, (2) fluffy non-woven inner fabric-heat-sealed nonwoven fabric PES 100%, (3) compacted non-woven inner fabric-compacted flat non-woven PP, (4) inner fabric-PA fabric, khaki dyed and (5) knitted fabric-PET 100% (Figure 1).

All textiles used for manufacturing the tested sleeping bags due to their complex internal structure were designed as a cuboid with physical features (density, thermal conductivity and specific heat) resulting from corresponding porosity showed in Table 2. Table 2 also presents the basic physical parameters of the raw materials from which the tested assembles are made.

#### 2.2.2. Physical Basis

Heat transfer simulations inside models of the tested sleeping bags were performed using finite volume method by SolidWorks Flow Simulation software [21]. The applied method can analyse the following physical phenomena: (1) heat transfer in solids (conduction); (2) free, forced and mixed convection; and (3) radiation both in the steady state and transient state [2,22,23,24,25,26,27]. A fuller description of the physical basis on which the modelling was carried out was demonstrated in an earlier work on modelling of the thermal performance of multilayer protective clothing exposed to radiant heat [27].

#### 2.2.3. Sleeping Bag Thermal Insulation Test

**Figure 2:**
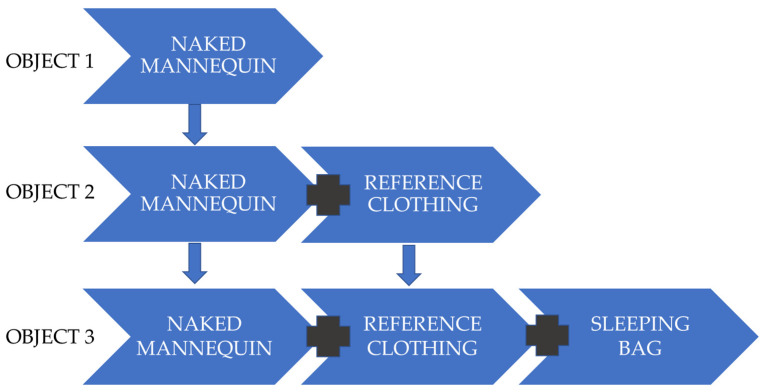
Graphical representation of the objects tested.

The main objective of the studies was to determine the thermal insulation of sleeping bags; however, in order to determine this, it was also necessary to check the loss of thermal energy in different thermal conditions of a naked mannequin and a mannequin in reference clothing. Only after completing these tests was it possible to proceed to the study of heat loss through the “reference clothing + sleeping bag” system (Figure 2).

**Figure 3:**
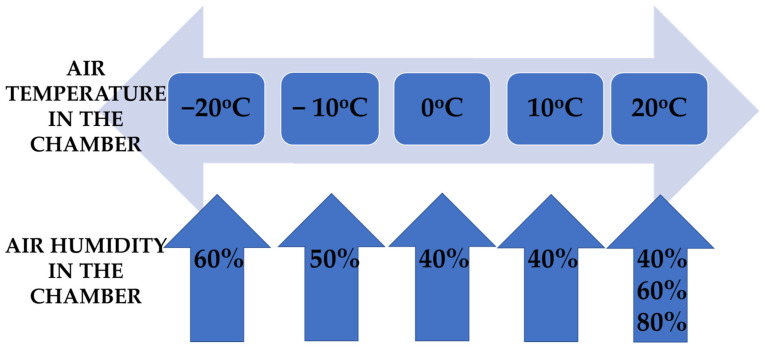
Graphical representation of the range of ambient conditions in the large chamber during testing.

The tests for each object were conducted at temperatures between −20 °C and 20 °C, and in the case of positive temperatures, the levels of relative humidity in the thermal chamber were also changed in the range of 40–80%. The temperatures of the tests were changed every 10°, while the humidity was changed every 20 percentage points (Figure 3). Each object was tested three times and the results from these measurements were then recorded and arithmetic averages were drawn. 

During the conduct of the tests, the mannequin was placed in a horizontal position on a sleeping pad characterised by thermal resistance of R_ct_ = (0.85 ± 0.06) m^2^·°C·W^−1^ tested in accordance with PN-EN ISO 11092:2014-11 and laid on a rigid surface. This surface consisted of a wooden plank meeting the requirements of ISO 1096 with dimensions, ensuring that the mannequin was fully positioned so that no part of the mannequin and the test object protruded beyond the plank, with a thickness of 20 mm ± 2 mm. The rigid surface was located 500 mm above the floor and the air circulation under the bench was provided. 

**Figure 4:**
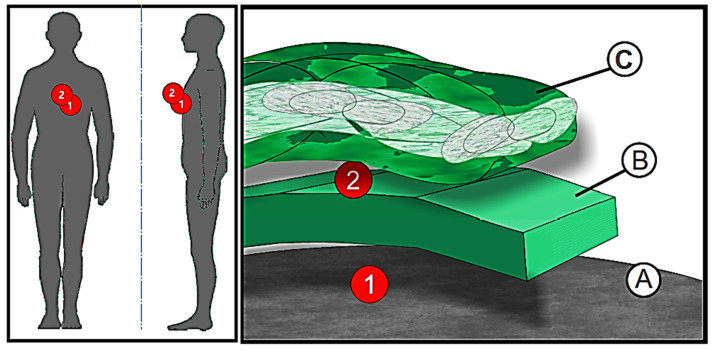
Graphical representation of the location of the temperature and humidity sensors: 1—Sensor between the body of the mannequin and the reference clothing; 2—Sensor over the reference clothing and under the sleeping bag; A—Body of the mannequin; B—Reference clothing; C—Sleeping bag.

Before the first test in the assumed climatic conditions, the tested objects were subjected to the acclimatization process in accordance with the ISO 139 standard for 24 h. Air parameters in under-clothing and inter-clothing layers were also tested. For this purpose, before each test, specially prepared temperature and humidity sensors were placed between each successive layer of clothing to check, without undressing the mannequin, what the conditions under a given clothing system were. These sensors were placed between (Figure 4):

− The body of the mannequin and the reference clothing (1);
− The reference clothing layer and the inner layer of the sleeping bag (2).

Both sensors were placed at the chest height of the mannequin. The sensors were continuously controlled by a computer program running the measuring modules. At the end of each test, the final temperature and humidity results from each module were recorded.

Prior to each subsequent test, the sleeping bags were acclimatised under constant atmospheric conditions for over 12 h. Once the air-conditioning chamber had been activated and the atmospheric conditions stabilised in the chamber, the appropriate clothing system was put on the mannequin in a manner that each tested item was put on the correct part of the body as it would be in normal use. In the case of sleeping bags, care was also taken to ensure that all components, such as zippers and drawstrings, were tightly closed—to reduce the spaces where heat could pass through from under the layers of fabric. In addition, sleeping bags were spread evenly over the body, loosening them beforehand. 

The mannequin dressed in a certain way and was placed horizontally on a sleeping pad in the centre of the climatic chamber, with its legs straight and its arms free at the sides of the mannequin’s body. Temperature and humidity sensors were placed under the respective layers of the material. The temperature of all body segments of the mannequin was set to maintain 35 °C. Air movement in the chamber was set at 0.4 m/s, while humidity and temperature were set according to the test cycle. The test result was recorded within 30 min, counting from the moment when the measurement conditions stabilised. Each day the tests were conducted in different air-conditioning conditions. 

#### 2.2.4. Air Permeability Test

In the transport of thermal energy through the clothing system, a large role is also played by the ventilation of the product; therefore, the tests for air permeability through the sleeping bag material were performed in order to check how the thickness of the insulation layer in the sleeping bag affects the air flow. The tests were carried out according to the PN-EN ISO 9237:1998 standard [28], using a Textest FX 3300-III device. The place of measurement on the fabric was chosen randomly, making sure that there were no seams or decorative and functional elements of the products in the measurement field and on its edges, which could influence the measurement. The device recorded the amount of air passing perpendicularly through the material; based on these results it calculated the air permeability R of the material expressed in mm/sec using the following formula [28]:

(6) $$R=\frac{\bar{q_{v}}}{A}\times 167$$

where $\bar{q_{v}}$—arithmetic mean of the quantity of air flowing through the material [dm^3^·min^−1^]; A—surface area of tested product [cm^2^]; 167—factor converting litres per minute into square centimetres per second.

#### 2.2.5. Maximum Sorption Test

**Figure 5:**
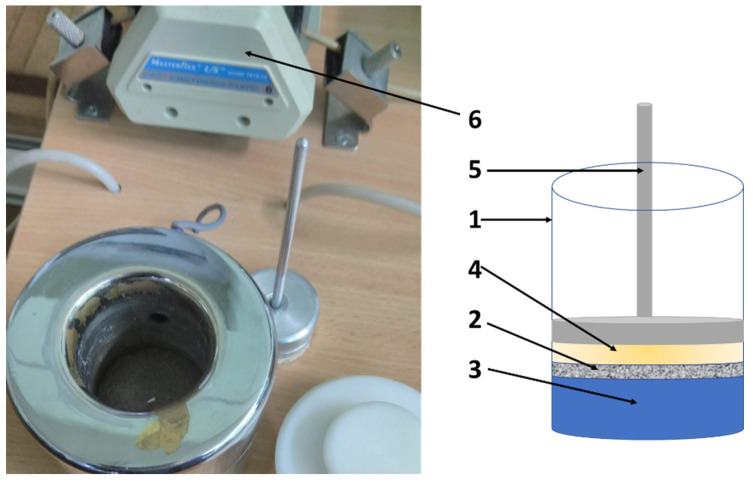
General view of the SORB 3 measuring instrument. 1—measurement chamber, 2—Schott plate, 3—regulating system sets the liquid level, 4—sample and 5—holder.

The study of physical phenomena that also have a significant impact on the shaping of biophysical properties of flat textile products includes the phenomenon of dynamic sorption. In order to examine the change in water vapour absorption capacity of flat textiles in time, an instrument called SORP 3 (Figure 5) was used. It is composed of measurement chamber 1 divided into two parts with the Schott plate 2. The regulating system sets the liquid level 3 in the upper part of the plate. The sample 4 is placed on the Schott plate with a holder 5, which, at the same time, forms the sample load. When the sample is inserted into the cup, a layer of water is absorbed through the pores of the sample and/or through the fibres. Under the Schott plate the water pressure decreases, which is registered by the pressure sensor. The control system starts the peristaltic pump 6, the task of which is to equalise the pressure under the plate. Based on the recording of the number of pump steps required to equalise the pressure, the volume of liquid that was absorbed by the test sample over time is calculated.

The instrument allows the recording of several parameters calculated based on the sorption kinetics curve chart, such as [7]: maximum sorption rate, described in µL-cm-s^−2−1^ as V_max_; maximum sorption value, designated in µL-cm^2^ as S_max_; initial time in *s* designated^-^ as t_0_; total sorption time in *s* designated as t_max_; sorption capacity designated as d.

The experiment performed was limited to the determination of the maximum sorption.

#### 2.2.6. Waterproofness Test

Tests of thermal insulation were carried out in various atmospheric conditions, including waterproofness of the external layer of sleeping bags. Before the tests, the samples were acclimatised in normal conditions for 24 h, in a free state, in accordance with the requirements included in the PN-EN ISO 139:2006/A1:2012 standard. The samples were taken from places without visible damage. The waterproofness test was carried out on an FX 3000 device according to PN-EN ISO 811:2018-07.

The research was conducted under the following conditions: surface area: 100 cm^2^, shape of the test surface: a circle, maximum test pressure: 2000 cm H_2_O, pressure increase speed: 60 cm H_2_O·min^−1^. Particular attention was paid to the fact that the visible side of the products should not have “water permeation points” or traces of moisture. A “water permeation point” is where a droplet has appeared like the prick of a pin and grows to a diameter of 1 mm to 1.5 mm. 

## 3. Results

### Conditions of Heat Transfer Simulations

**Figure 6:**
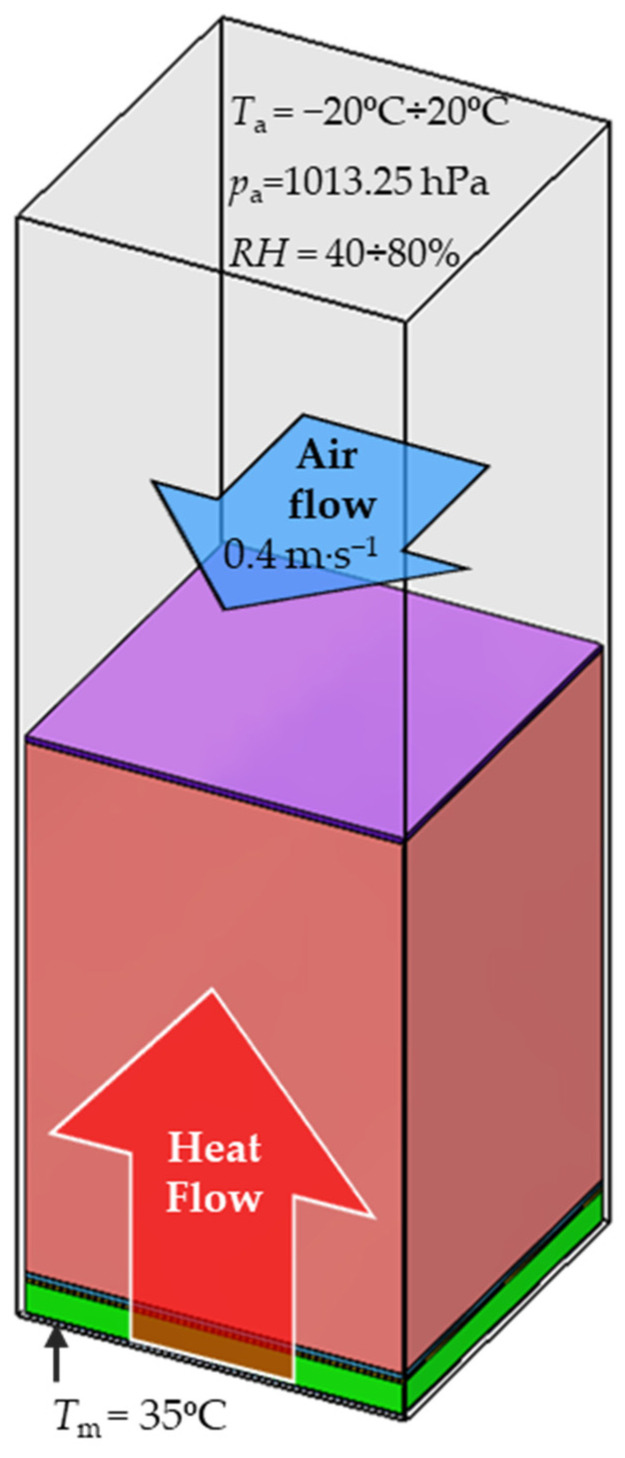
Conditions of the heat transfer simulations.

The main aim of the heat transfer simulations was to determine thermal insulation of two tested material arrangement of sleeping bags in the different environmental conditions, in which the thermal insulation of real assembles was measured using the thermal mannequin. For this purpose, each of the two models was placed on a plate model with a constant temperature of 35 °C. The assembly model and plate model were situated inside a rectangular computing domain, 30 mm high, filled by air (Figure 6). 

Conditions inside the computational domain were consistent with the initial conditions of the thermal resistance measurement, i.e., T_a_ = −20 °C ÷ 20 °C, p_a_ = 1013.25 hPa, RH = 40% ÷ 80%, temperature heat plate T_m_ = 35 °C, horizontal airflow speed 0.4 m·s^−1^. As a result of the temperature difference between the heat plate and the surroundings, a heat transfer occurs through the sleeping bag model in a vertical upward direction. As a result of heat transfer, successive layers of the sleeping bag model heat up, reaching a certain constant temperature after reaching a steady state.

**Figure 7:**
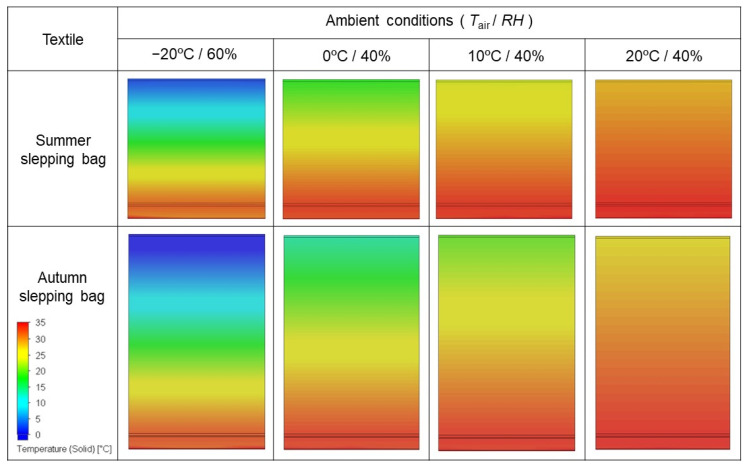
Temperature distributions on cross-sections of the tested sleeping bag models.

To imitate an infinite three layers of assembly propagating outside of computational domain in all four horizontal directions, periodic boundary conditions were applied. The computational domain was divided into three types of cells: solid, gas and partial. The number of cells was different for each sleeping bag model due to their geometry (Table 3). Temperature distributions on cross-sections of the tested sleeping bags models are presented in the Figure 7.

**Figure 8:**
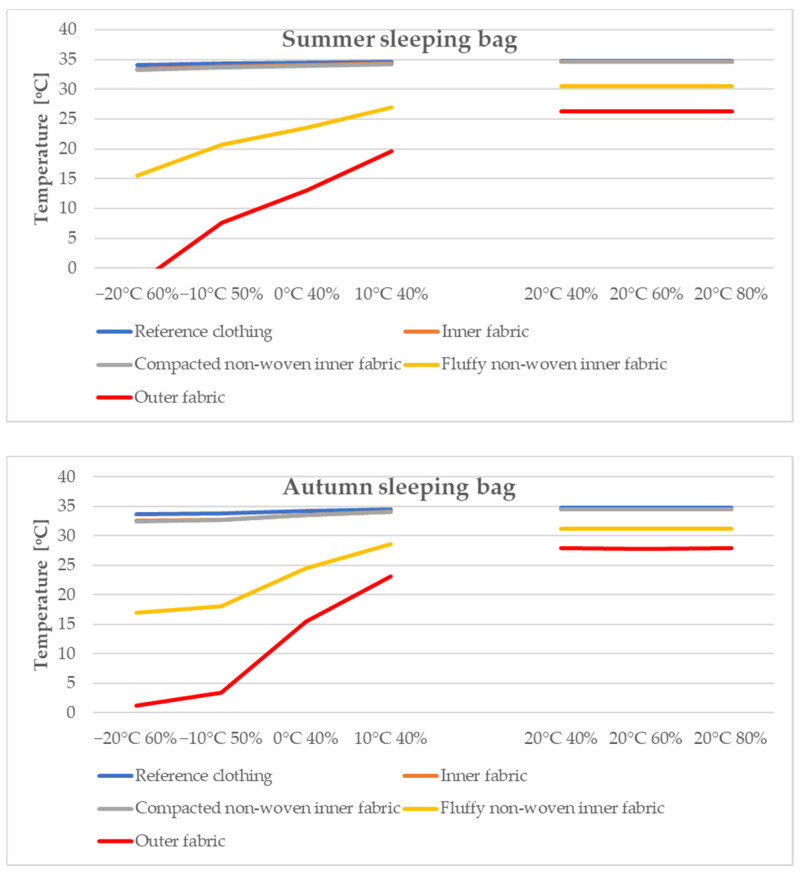
Summary of average temperatures obtained during computer simulations in individual zones of the tested sleeping bags.

As a result of the simulations, the temperature ranges in the individual components of the sleeping bags and reference clothing in various climatic conditions (after reaching the steady state) were determined. Figure 8 summarises the average temperatures obtained during the computer simulations.

Based on the analysis of the data presented in Figure 8, it can be observed that with the increase of the temperature difference between the panel model, the temperature of which was constant (35 °C) and the external temperature in the external layers of the materials, the temperature decreased significantly. In the case of layers close to the heat source, this temperature changed slightly because it is protected by the outer layers. The conducted research also allows us to observe that the temperature changes in subsequent layers of sleeping bags are slightly greater for summer sleeping bags than for winter ones, which results from the thickness of the insulation placed inside them.

According to Fourier’s law, the rate of heat transfer through a material is directly proportional to the cross-sectional area from which the heat passes and the temperature difference along the end surfaces of the material. According to the experiment carried out and the results presented in Table 4, it can be concluded that the heat transfer coefficient (W) increases with the increase of the temperature difference between the heating plate and the environment. A constant thermal conductivity and cross-sectional area equal 1 m^2^ of the simulated research material were assumed for the calculations. The performed simulations confirm the correctness of the operation in accordance with Fourier’s law.

As a result of the performed tests at the assumed temperatures and humidity, the following values were determined:

- Total heat insulation for the tested clothing sets;
- Temperature and humidity between the body of the mannequin, clothing system and between the layers of the clothing system;
- Air permeability of sleeping bags.

Prior to testing the clothing sets, a naked mannequin was placed in an environmental chamber in the supine position described in the methodology to determine its thermal insulation over the climate ranges, as shown in Figure 2. 

Prior to each subsequent test, the sleeping bags were acclimatised under constant atmospheric conditions for over 12 h. Once the air-conditioning chamber has been activated and the atmospheric conditions stabilised in the chamber, the appropriate clothing system was put on the mannequin in a manner that each tested item was put on the correct part of the body, as it would be in normal use. The test result was recorded within 30 min, counting from the moment when the measurement conditions stabilised. Each day the tests were conducted in different air-conditioning conditions. Each object was tested three times and the results from these measurements were then recorded and arithmetic averages were drawn. In temperatures below 0 °C, due to technological limitations, it was not possible to determine the prevailing air humidity in the chamber, although the humidity values were read by sensors inside the chamber used for monitoring the environmental conditions. The results of the total thermal insulation of the naked mannequin are summarised in Table 5. After the testing of the naked mannequin, the thermal insulation power of the mannequin dressed in type B reference clothing complying with PN-EN 342:2018-01 was tested.

Based on the analysis of the data presented in Table 5, it can be observed that as the temperature increases, the thermal insulation of the naked mannequin increases. This is because the energy flux required to be delivered to the mannequin is lower at positive temperatures to maintain the surface of the mannequin at a constant thermal level. The table demonstrates that when the mannequin was dressed in the reference clothing, the thermal insulation increased by an average of 0.0 06 m^2^·°C ·W^−1^ at a constant humidity level of 40% for both 10 °C and 20 °C temperature changes. The effective thermal insulation of the reference clothing behaved identically to that of the naked mannequin—an increase of the temperature resulted in an increase in thermal insulation and a change in humidity to a higher level resulted in a small change in final thermal insulation within the measurement error range. 

After testing the thermal insulation of the reference clothing, the test of mannequin–reference clothing–sleeping bag systems was commenced.

**Figure 9:**
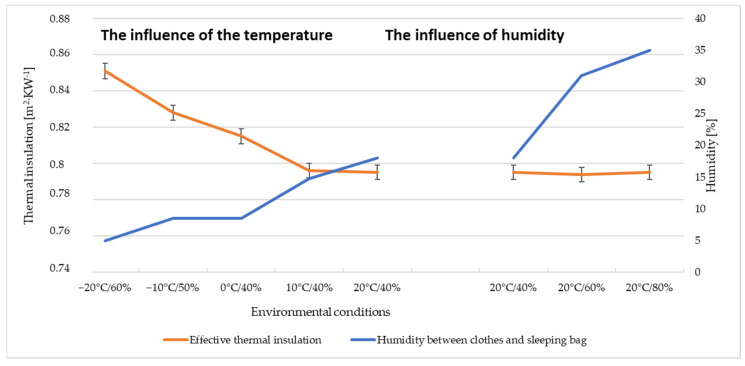
Thermal insulation of a mannequin in a summer sleeping bag.

When analysing the test results specified in Table 6 and Figure 9, it can be observed that the effective thermal insulation of the summer sleeping bag increased in the range of 0.60 ÷ 0.68 m^2^·°C·W^−1^ compared to the thermal insulation of the reference clothing. When the summer sleeping bag is added to the clothing system, the effective thermal insulation starts to decrease with increasing temperature. Figure 9 shows the dependence of thermal insulation on the moisture content measured under the clothing layers (Table 7). It shows that when the moisture content under the clothing increases, the effective thermal insulation of the summer sleeping bag remains at the same level.

**Figure 10:**
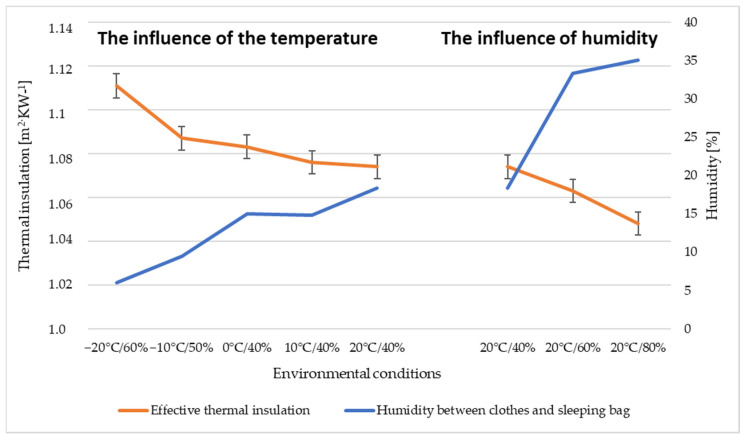
Thermal insulation of a mannequin in an autumn sleeping bag.

The effective thermal insulation of the clothing system, after the addition of the autumn sleeping bag (Table 8), increased in the range of 0.85 ÷ 0.94 m^2^·°C·W^−1^ compared to the reference clothing system. As in the case of the summer sleeping bag, the effective thermal insulation decreases with increasing temperature. Figure 10 shows the dependence of the thermal insulation on the moisture content measured under the clothing layers (Table 7), from which as the moisture content under the clothing increases, and the effective thermal insulation of the autumn sleeping bag slightly decreases.

When testing the thermal insulation of sleeping bags, temperature and humidity sensors were placed between the layers of clothing to see what conditions prevailed between each layer after being exposed to constant climatic conditions for several hours. At the end of each test in each atmosphere set in a climate chamber, the results from the sensors were recorded for later comparison.

In Table 7, the results of the environmental conditions set in the large-size chamber and the values of temperature and humidity measured under clothing layers are juxtaposed to compare the influence of these factors on the behaviour of the clothing system. It can be observed that the temperature between the layers of the clothing system increased when the temperature of the external environment increased. As can also be observed, the temperature between clothing layers increased twice as fast in negative temperatures than the temperature at the body. 

**Figure 11:**
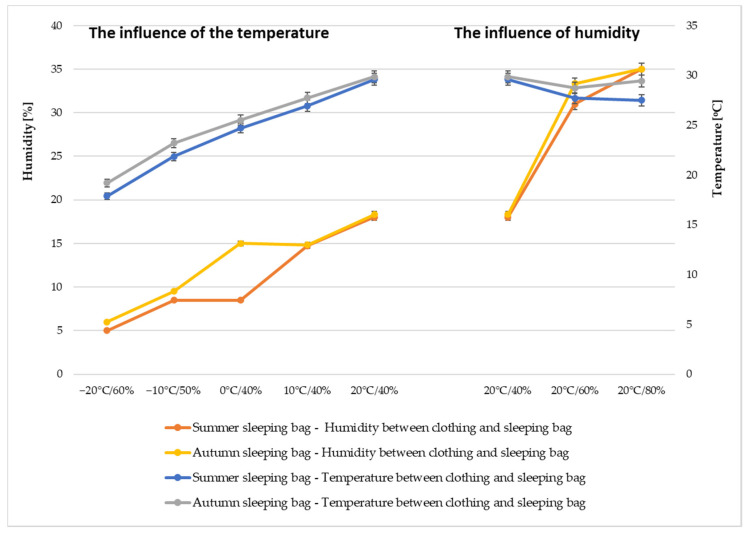
Average humidity and temperature obtained between the clothing and the sleeping bag under different weather conditions.

Figure 11 shows how the humidity in the clothing system increased as the outdoor humidity changed. When the outdoor temperature was negative and the outdoor humidity was in the range of 50–60%, the underclothing humidity remained in the range of 5–10%. When the tests were performed with a constant temperature of 20 °C and only the external humidity was changed in the range of 40–80%, the changes in humidity in the underclothing layers were only in the range of 18–35%. Considering that the sleeping bags have a waterproof finish in their outer layer, it can be concluded that the amount of moisture found in the surroundings of the test object is let through to the inside of the sleeping bag in a small amount. 

After the tests in the environmental chamber, both sleeping bags were subjected to the air permeability test, making sure that no additional elements of the sleeping bags are in the outlet nozzle of the measuring apparatus. The mean, together with the standard deviation and the coefficient of variation, was drawn from the results obtained. The results are presented in Table 9. 

When analysing the data specified in Table 9, it can be observed that the autumn sleeping bag has a marginally lower air permeability. The slight differences are dictated by the fact that both sleeping bags were made of the same external materials. They only differ in the fleece insert and construction.

In addition, tests of the components of the sleeping bag were performed to determine the maximum sorption associated with biophysical comfort and waterproofness. The results are presented in Table 10.

Based on the performed thermal insulation tests, the temperature ranges, in which the tested sleeping bags can be used, were determined. According to PN-EN ISO 23537-1:2017-02, the following temperatures were determined:

− Comfort temperature (T_comf_) is the lower limit of the comfort range up to which a sleeping bag user in a relaxed posture, e.g., lying on his or her back, is in thermal equilibrium and simply does not feel cold. (The data for this temperature consists of: (a) basic metabolic heat production: M_b_ = 44.4 W/m^2^; (b) additional metabolic heat production due to shivering: M_s_ = 25.4 W/m^2^; (c) effective thermal resistance of the sleeping bag R_c,eff_:R_c,eff_ = R_c_; (d) effective water vapour resistance of the sleeping bag R_e,eff_:R_e,eff_ = 60 R_c,eff_/0.54);
− Limit temperature (T_lim_) is the lower limit at which the user of a sleeping bag with the body rolled up is in thermal equilibrium and simply does not feel cold (The data for this temperature consists of: (a) basic metabolic heat production: M_b_ = 47.5 W/m^2^; (b) effective thermal resistance of the sleeping bag R_c,eff_:R_c,eff =_ R_c_; (c) effective water vapour resistance of the sleeping bag R_e,eff_:R_e,eff_ = 60 ⋅ R_c,eff/0.54_);
− Extreme temperature (T_ext_) is a very low temperature at which there is a risk of damage to health by hypothermia (g, 1.60 m, 1.62 m^2^ body surface area), which is just not feeling cold (no shivering) in a relaxed posture. The data for this temperature consists of: (a) basic metabolic heat production: M_b_ = 44.4 W/m^2^; (b) effective thermal resistance of the sleeping bag R_c,eff_:R_c,eff_ = 0.9 R_c_; (c) effective water vapour resistance of the sleeping bag R_e,eff_:R_e,eff_ = 60 ⋅ R_c,eff_/0.5) [8].

**Figure 12:**
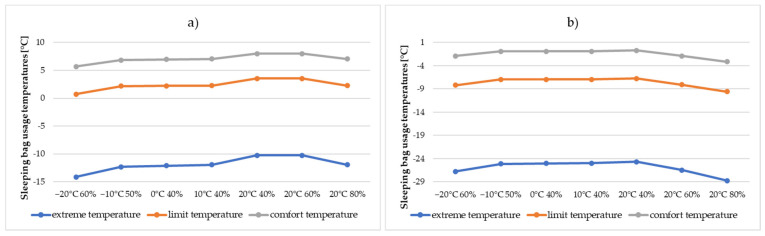
Comparison of lower temperatures of sleeping bags in relation to the environmental conditions of the tests conducted for (**a**) summer sleeping bags and (**b**) autumn sleeping bags.

When analysing the data summarised in Table 11 and Figure 12, it can be observed that the extreme temperature for the summer sleeping bag, depending on the environmental conditions in which the test was conducted, varies from—10.24 °C to −14.15 °C, while for the autumn sleeping bag, varies from −24.66 °C to −28.75 °C. For the autumn sleeping bag, the biggest differences are observed in the case of comfort temperature, which ranges from −0.71 °C ÷ −3.13 °C, while for the summer sleeping bag, ranges from 5.66 °C to 7.99 °C. The limit temperature in which the autumn sleeping bag can be used ranges from −6.74 °C to −9.58 °C and summer sleeping bag in the range of 0.71 °C to 3.55 °C. It should be observed, however, that the temperature differences for both types of sleeping bags, depending on the assumed external ambient conditions, do not exceed 4 °C.

## 4. Discussion

The presented research results demonstrate the complexity of the process of measuring the thermal insulation of clothing products. It is influenced by temperature and air humidity. The thermal insulation of products protecting against cold also changes depending on the structure and layering of clothing systems of which a given textile product is made. In this case, the insulation values of individual layers of systems do not add up but are a resultant of many parameters of a given environment. The final thermal insulation of a given system, apart from the layering of the system, is also influenced by the amount of air between the layers and its parameters as well as the structure of the textiles in each layer and the morphological parameters of the fibres in these materials. 

After conducting simulations using SolidWorks, which served as a theoretical model, the thermal insulation properties of sleeping bags were verified in simulated conditions on a thermal mannequin placed in a large-size chamber. The simulations carried out allowed us to formulate a thesis stating that when using the same thermal conditions with changing ambient humidity conditions, thermal insulation should remain at a constant level. In the case of temperature changes, the thermal insulation will increase with the increase of the temperature gradient.

According to Figure 8 and Table 4 for summer sleeping bags, the temperature gradient between the outer layers of the tested system under the conditions of −20 °C/60% was −36.69 °C·m^−1^ (thermal conductivity coefficient 21.42 W), and for the conditions of 20 °C/40% −8.46 °C·m^−1^ (21.42 W). In the case of autumn sleeping bags, both the temperature gradient and the thermal conductivity coefficient were lower. For tests in the conditions of 0 °C/40%, for the autumn sleeping bag, the temperature gradient was −18.81 °C·m^−1^ m (heat conduction coefficient—10.90 W), and for the summer sleeping bag it was −21.43 °C·m^−1^ (12.50 W), respectively.

On the basis of the data presented in Table 5, it was observed that the thermal insulation of the naked mannequin increases along with the increase of temperature. Changes in the ambient humidity level at the same temperature of 20 °C do not significantly affect the thermal insulation value of the mannequin’s body surface. A similar situation occurs in the case of the effective insulation of the reference clothing, which adhered to the mannequin’s body, forming a thin protective layer on its surface against external environmental factors. 

For sleeping bags, the effective thermal insulation decreases along with the increase of temperature and decrease of humidity. When analysing the data of effective thermal insulation for a winter sleeping bag, it can be observed that for a temperature of 20 °C and humidity of 60%, thermal insulation is 1.063 m^2^·°C·W^−1^, while for a temperature of −20 °C and humidity of 60%, thermal insulation increases significantly and is 1.111 m^2^·°C·W^−1^. A similar situation occurs for the effective thermal insulation of a summer sleeping bag (20 °C/60% thermal insulation is 0.794 m^2^·°C·W^−1^, while for −20 °C/60%—0.851 m^2^·°C·W^−1^.

If we maintain the temperature constant at 20 °C and only change the ambient humidity between 40% and 80%, it can be observed that the changes in the effective thermal insulation of summer sleeping bags are at a constant level and do not change.

For autumn sleeping bags, the effective thermal insulation decreases along with the increase of humidity for a temperature of 20 °C (at 40% humidity—1.074 m^2^·°C·W^−1^ and for 80%—1.048 m^2^·°C·W^−1^). 

In addition, it can be observed that the temperature between clothing layers increases along with the increase of temperature of the external environment. When the measurements were conducted at the temperature of −20 °C and humidity of 60%, the temperature between the clothing and the summer sleeping bag was 17.87 °C, while for the conditions of 20 °C^/^60%—27.70 °C. This may be because the outer fabric has a high degree of waterproofness at 714.28 ± 5 mm H_2_O and a low sorption level of 1.26 ± 0.2 μL·cm^−2^, which minimally allows moisture to penetrate the sleeping bag and the mannequin itself. 

The differences in effective thermal insulation may result from changes in humidity under the clothing by approximately 17% when the external humidity changes were at the level of 40% and is well illustrated by the sensor measurement between the clothing and the autumn sleeping bag, which for the test conditions of 20 °C^/^40% was 18.33%, while for 20 °C^/^80% it amounted to 35.00%. It was also observed that along with the increase of external humidity, the underclothing temperature remained at a similar thermal level. Thus, differences in humidity levels between the system and the external environment can be a parameter that acts as additional insulation against heat loss through the body. 

Comparing the insulation data of all systems, it can be concluded that the final thermal insulation, in addition to atmospheric conditions, is also affected by the thickness of the clothing system insulating the user’s body from the external environment, and the air between the layers of the sleeping bag is of great importance.

## 5. Conclusions

The conducted experiment allows us to conclude that before starting the actual tests with the use of specialised and unique equipment, such as a thermal mannequin, it is advisable to conduct simulation tests for layered systems, such as heat-insulating clothing or sleeping bags. In the case of layered systems, the clove’s thermal insulation is influenced not only by external conditions, but also by the number of component layers, raw material composition, physical properties and many other features. Until now, publications related to the analysis of thermal insulation of various one or two-layer systems occurred in literature; however, the examination was always held in constant thermal conditions. The authors attempted to determine the influence of variable climatic conditions on the obtained thermal insulation properties of the multilayer systems. Taking into account the obtained results, it can be concluded that the effective thermal insulation of the tested sleeping bags changes under the influence of an air temperature increase. When comparing the data on the effective thermal insulation of sleeping bags, one can see a correlation between the increase in effective thermal insulation and the increase in the temperature difference between the mannequin and the external environment. In the case of a mannequin dressed only in reference clothing, an increase in effective thermal insulation is observed along with the decreasing temperature difference between the environment and the mannequin. In the case of multilayer clothing (clothing system with sleeping bag), the effective thermal insulation increases along with the increase of temperature difference between the mannequin and the external environment. The sleeping bags are made of fabric with a waterproof finish with limited air permeability; thus, the effective thermal insulation is much higher than that of a single-layer system—the reference clothing. Both summer and autumn sleeping bags meet the military requirements. At the test temperature of 10 °C required by EN ISO 23537-1, they achieve an effective thermal insulation of over 0.780 m^2^·°C·W^−1^—summer sleeping bag and over 1.021 m^2^·°C·W^−1^—autumn sleeping bag. 

As part of further work related to the assessment of thermal insulation, the authors will undertake work related to the assessment of this parameter for multilayer systems with constant climatic conditions and variable raw material systems.

## Figures and Tables

**Table 1:** Basic data on the weight of sleeping bags.

Surface Weight of the Sleeping Bag [g·m^−2^] (Coefficient of Variation [%])	Summer Sleeping Bag 1.2 kg (3%)	Autumn Sleeping Bag 1.8 kg (3%)	Raw Material Layer Composition
Outer fabric	66 ± 4	66 ± 4	PA fabric, khaki dyed with panther print and waterproof finish
Fluffy non-woven inner fabric	60 ± 5	133 ± 7	Heat-sealed nonwoven fabric PES 100%
Compacted non-woven inner fabric	15 ± 1	15 ± 1	Compacted flat non-woven PP
Inner fabric	57 ± 3	57 ± 3	PA fabric, khaki dyed

**Table 2:** Physical features of the raw materials applied in simulations.

		Physical Parameter			
Surface	Raw Material	Density [kg·m^−3^]	Specific Heat [J·kg^−1^·°C^−1^]	Thermal Conductivity [W·m^−1^·°C^−1^]	Porosity [%]
Reference clothing	PET knitted fabric	1370	1380	0.08	39
Outer fabric	PA fabric, khaki dyed with panther print and waterproof finish	1230	2050	0.22	19
Fluffy non-woven inner fabric	Heat-sealed nonwoven fabric PET	1370	1380	0.084	61
Compacted non-woven inner fabric	Compacted flat non-woven PP	940	1720	0.26	61
Inner fabric	PA fabric, khaki dyed	1230	2050	0.22	20
Air	-	1.2	1005	0.03	-

**Table 3:** Number of cells in 3D models of the tested sleeping bags.

Model	Solid Cells	Gas Cells	Partial Cells
Summer sleeping bag	8232	1480	532
Autumn sleeping bag	5880	1684	484

**Table 4:** Summary of the thermal conductivity coefficient and the temperature gradient of the tested sleeping bags.

Air Temperature [°C]	Air Humidity [%]	Established Thermal Conductivity Coefficient [W·m^−1^·°C^−1^]	Temperature Differences Between the Outer Fabric and the Reference Clothing [°C·m^−1^]	Heat Transfer Coefficient [W]
			Summer sleeping bag	
−20	60	0.584	−36.69	21.42
−10	50	0.584	−26.73	15.59
0	40	0.584	−21.43	12.50
10	40	0.584	−15.09	8.76
20	40	0.584	−8.46	4.94
20	60	0.584	−8.46	4.94
20	80	0.584	−8.46	4.94
			Autumn sleeping bag	
−20	60	0.584	−32.50	18.89
−10	50	0.584	−30.43	17.75
0	40	0.584	−18.81	10.90
10	40	0.584	−11.42	6.70
20	40	0.584	−6.87	3.97
20	60	0.584	−6.87	3.97
20	80	0.584	−6.87	3.97

**Table 5:** Total thermal insulation of the naked mannequin and the reference clothing under different environmental conditions.

Air Temperature [°C]	Air Humidity [%]	Thermal Insulation of the Naked Mannequin [m^2^·°C ·W^−1^]	Thermal Insulation of Reference Clothing [m^2^·°C ·W^−1^]	
			Total	Effective
−20	60	0.100 ± 0.003	0.271 ± 0.004	0.171 ± 0.003
−10	50	0.101 ± 0.003	0.304 ± 0.004	0.179 ± 0.003
0	40	0.106 ± 0.003	0.311 ± 0.004	0.184 ± 0.004
10	40	0.113 ± 0.003	0.324 ± 0.004	0.190 ± 0.004
20	40	0.120 ± 0.003	0.310 ± 0.004	0.190 ± 0.004
20	60	0.121 ± 0.003	0.313 ± 0.004	0.192 ± 0.003
20	80	0.122 ± 0.003	0.315 ± 0.004	0.193 ± 0.003

**Table 6:** Average thermal insulation of a mannequin in a summer sleeping bag.

Air Temperature [°C]	Air Humidity [%]	Thermal Insulation [m^2^·°C ·W^−1^] Summer Sleeping Bag	
		Total	Effective
−20	60	0.951 ± 0.004	0.851 ± 0.004
−10	50	0.929 ± 0.004	0.828 ± 0.004
0	40	0.921 ± 0.004	0.815 ± 0.003
10	40	0.908 ± 0.004	0.796 ± 0.003
20	40	0.915 ± 0.004	0.795 ± 0.004
20	60	0.915 ± 0.004	0.794 ± 0.004
20	80	0.917 ± 0.003	0.795 ± 0.003

**Table 7:** Average temperature and humidity measured under the clothing layers of summer and autumn sleeping bags under different weather conditions.

Parameters Set in the Chamber	Parameters Recorded with Sensors				
	Location of Sensors	Summer Sleeping Bag		Autumn Sleeping Bag	
		Air Temperature [°C]	Air Humidity [%]	Air Temperature [°C]	Air Humidity [%]
−20 °C 60%	Between body of the mannequin and clothing	28.80 ± 0.15	7.67 ± 1.20	29.10 ± 0.01	7.80 ± 0.01
	Between clothing and sleeping bag	17.87 ± 0.64	5.00 ± 0.79	19.20 ± 0.01	6.00 ± 0.01
−10 °C 50%	Between body of the mannequin and clothing	29.33 ± 0.01	10.00 ± 0.41	30.80 ± 0.01	10.50 ± 0.41
	Between clothing and sleeping bag	21.85 ± 0.07	8.50 ± 0.42	23.20 ± 0.01	9.50 ± 0.42
0 °C 40%	Between body of the mannequin and clothing	31.20 ± 0.07	10.00 ± 1.28	32.10 ± 0.16	16.50 ± 1.22
	Between clothing and sleeping bag	24.70 ± 0.62	8.50± 0.66	25.50 ± 0.13	15.00 ± 0.81
10 °C 40%	Between body of the mannequin and clothing	33.63 ± 0.62	13.75 ± 0.29	34.10 ± 0.10	15.67 ± 1.20
	Between clothing and sleeping bag	26.95 ± 0.04	14.75 ± 0,55	27.73 ± 0.03	14.83 ± 1.33
20 °C 40%	Between body of the mannequin and clothing	34.80 ± 0.01	18.00 ± 0.01	36.07 ± 0.03	19.33 ± 0.33
	Between clothing and sleeping bag	29.60 ± 0.02	18.00 ± 0.01	29.87 ± 0.03	18.33 ± 0.33
20 °C 60%	Between body of the mannequin and clothing	33.90 ± 0.01	29.00 ± 0.01	36.13 ± 0.03	29.33 ± 1.20
	Between clothing and sleeping bag	27.70 ± 0.01	31.00 ± 0.01	28.73 ± 0.66	33.33 ± 0.88
20 °C 80%	Between body of the mannequin and clothing	34.33 ± 0.01	35.00 ± 0.01	34.50 ± 1.22	35.06 ± 1.63
	Between clothing and sleeping bag	27.50 ± 0.03	35.00 ± 0.01	29.45 ± 0.88	35.00 ± 1.29

**Table 8:** Average thermal insulation of a mannequin in an autumn sleeping bag.

Air Temperature [°C]	Air Humidity [%]	Thermal Insulation [m^2^·°C·W^−1^] Autumn Sleeping Bag	
		Total	Effective
−20	60	1.211 ± 0.004	1.111 ± 0.004
−10	50	1.188 ± 0.004	1.087 ± 0.004
0	40	1.189 ± 0.004	1.083 ± 0.004
10	40	1.189 ± 0.003	1.076 ± 0.004
20	40	1.194 ± 0.004	1.074 ± 0.005
20	60	1.184 ± 0.004	1.063 ± 0.004
20	80	1.170 ± 0.004	1.048 ± 0.004

**Table 9:** Average air permeability of both sleeping bags.

	Air Permeability [mm/s]	Standard Deviation [mm/s]	Coefficient of Variation [%]
Summer sleeping bag	0.694	0.421	60.663
Autumn sleeping bag	0.516	0.212	41.085

**Table 10:** Test results of sorption and waterproof properties.

Components of the Sleeping Bag	Maximum Sorption [μl cm^−2^]	Waterproofness [mmH_2_O]	Raw Material Composition of the Layer
Outer fabric	1.26 ± 0.2	714.28 ± 5	PA fabric, khaki dyed, with panther print and waterproof finish
Fluffy non-woven inner fabric	9.57 ± 0.3	permeation	Heat-sealed nonwoven fabric PES 100%
Non-woven inner fabric	8.43 ± 0.3	permeation	Compacted flat non-woven PP
Inner fabric	3.69 ± 0.2	357.14 ± 4	PA fabric, khaki dyed

**Table 11:** Lower temperature limits in the range of utility.

Type of Sleeping Bag	Environmental Conditions	Thermal Insulation [m^2^·°C·W^−1^]	Extreme Temperature (T_ext_) [°C].	Limit Temperature (T_lim_) [°C].	Comfort Temperature (T_comf_) [°C].
Summer sleeping bag	−20 °C/60%	0.851	−14.150	0.707	5.659
	−10 °C/50%	0.828	−12.319	2.179	6.833
	0 °C/40%	0.815	−12.126	2.213	6.942
	10 °C/40%	0.804	−11.962	2.244	7.037
	20 °C/40%	0.791	−10.242	3.550	7.987
	20 °C/60%	0.791	−10.242	3.550	7.987
	20 °C/80%	0.804	−11.962	2.244	7.037
Autumn sleeping bag	−20 °C/60%	1.111	−26.765	−8.181	−1.919
	−10 °C/50%	1.087	−25.124	−6.971	−0.918
	0 °C/40%	1.083	−25.032	−6.945	−0.915
	10 °C/40%	1.081	−24.985	−6.933	−0.914
	20 °C/40%	1.067	−24.662	−6.744	−0.705
	20 °C/60%	1.099	−26.476	−8.093	−1.898
	20 °C/80%	1.15	−28.750	−9.583	−3.127
